# Hypericin, a potential new BH3 mimetic

**DOI:** 10.3389/fphar.2022.991554

**Published:** 2022-10-04

**Authors:** Anastasia Doroshenko, Silvia Tomkova, Tibor Kozar, Katarina Stroffekova

**Affiliations:** ^1^ Department of Biophysics, Faculty of Natural Sciences, PJ Safarik University, Kosice, Slovakia; ^2^ Center of Interdisciplinary Biosciences, TIP-Safarik University, Kosice, Slovakia

**Keywords:** Bcl-2 proteins, BH3 mimetics, hypericin, gossypol, cancer

## Abstract

Many types of cancer such as prostate cancer, myeloid leukemia, breast cancer, glioblastoma display strong chemo resistance, which is supported by enhanced expression of multiple anti-apoptotic Bcl-2, Bcl-XL and Mcl-1 proteins. The viable anti-cancer strategies are based on developing anti-apoptotic Bcl-2 proteins inhibitors, BH3 mimetics. Our focus in past years has been on the investigating a new potential BH3 mimetic, Hypericin (Hyp). Hyp is a naturally occurring photosensitive compound used in photodynamic therapy and diagnosis. We have demonstrated that Hyp can cause substantial effects in cellular ultrastructure, mitochondria function and metabolism, and distribution of Bcl2 proteins in malignant and non-malignant cells. One of the possible mechanisms of Hyp action could be the direct interactions between Bcl-2 proteins and Hyp. We investigated this assumption by in silico computer modelling and *in vitro* fluorescent spectroscopy experiments with the small Bcl2 peptide segments designed to correspond to Bcl2 BH3 and BH1 domains. We show here that Hyp interacts with BH3 and BH1 peptides in concentration dependent manner, and shows the stronger interactions than known BH3 mimetics, Gossypol (Goss) and ABT-263. In addition, interactions of Hyp, Goss and ABT263, with whole purified proteins Bcl-2 and Mcl-1 by fluorescence spectroscopy show that Hyp interacts stronger with the Bcl-2 and less with Mcl-1 protein than Goss or ABT-263. This suggest that Hyp is comparable to other BH3 mimetics and could be explore as such. Hyp cytotoxicity was low in human U87 MG glioma, similar to that of ABT263, where Goss exerted sufficient cytotoxicity, suggesting that Hyp acts primarily on Bcl-2, but not on Mcl-1 protein. In combination therapy, low doses of Hyp with Goss effectively decreased U87 MG viability, suggesting a possible synergy effect. Overall, we can conclude that Hyp as BH3 mimetic acts primarily on Bcl-2 protein and can be explored to target cells with Bcl-2 over-expression, or in combination with other BH3 mimetics, that target Mcl-1 or Bcl-XL proteins, in dual therapy.

## Introduction

Programmed cell death (apoptosis) is a particularly conserved process that maintains healthy cell populations within tissues. Apoptosis is tightly regulated and disrupting its control mechanisms is the underlying cause of cancer initiation and expansion. B-cell lymphoma-2 (Bcl-2) family of proteins are the main regulators of apoptosis (Vaux and Korsmeyer, 1999; Danial and Korsmeyer, 2004). The Bcl-2 family consists of multi BH domain anti- (Bcl-2, Bcl-XL, Mcl-1, Bfl-1, Bcl-W, and Bcl-B) and pro-apoptotic members (Bax, Bak and Bok). The Bcl-2 family also contains a group of BH3-only proteins (Bad, Bim, Bmf, Bik, Hrk, Bid, Puma, Noxa), which can serve as sensitizers or direct activators of apoptosis (Cory et al., 2003; Llambi et al., 2011). The balanced system of protein-protein interactions between multi BH domain anti- and pro-apoptotic Bcl-2 proteins, and/or BH3-only proteins manages either cell survival or death (Llambi et al., 2011). A cell’s ability to undergo apoptosis strongly depends on the presence of BH3-only proteins and their interaction with anti-apoptotic members.

Cancer cells escape apoptosis by the upregulating of anti-apoptotic Bcl2 proteins (Bcl2, Bcl-XL and Mcl1), and this upregulation significantly adds to tumor progression, poor prognosis, and chemo resistance (Vogler, 2014; Correia et al., 2015; Gong et al., 2016). Thus, these proteins and their interactions represent attractive targets for new anticancer treatments (Davids and Letai, 2012; Vela and Marzo, 2015). The Bcl-2 proteins structural studies (X-ray crystallography and NMR) revealed a hydrophobic groove comprised of BH3, BH2 and BH1 domain on the surface of anti-apoptotic Bcl-2 family proteins that binds the BH3 domain of their pro-apoptotic partners (Petros et al., 2004). Based on this interaction, different strategies have been employed. There were studies of peptides that mimic BH3 motifs of pro-apoptotic Bak, Bax or BH3-only proteins (Wang et al., 2000a; DeBartolo et al., 2012; Raghav et al., 2012), or use of small molecules that inhibit pro-survival Bcl2 proteins (Wang et al., 2000b; O’Neill et al., 2004; Wang et al., 2006; Azmi and Mohammad, 2009; Davids and Letai, 2012). More than 25 small molecules, termed BH3 mimetics, with diverse chemical structures were designed as inhibitors of anti-apoptotic Bcl2 proteins (Bcl2, BclXL and Mcl1), and are currently used in preclinical and clinical studies (Wang et al., 2000a; Wang et al., 2000b; O’Neill et al., 2004; Wang et al., 2006; Azmi and Mohammad, 2009; Davids and Letai, 2012; Raghav et al., 2012; Vela and Marzo, 2015). The available BH3 mimetics are either naturally occurring (Gossypol, Chelerythrine, Antimycin A) or artificially synthetized (HA14-1, ABT 263, BI-21C6), and it is very likely that a number of new BH3 mimetics will appear (Tzung et al., 2001; Chmura et al., 2000; Oltersdorf et al., 2005; Rega et al., 2007; Trudel et al., 2007; Tse et al., 2008; Azmi and Mohammad, 2009; Voss et al., 2010; Lian et al., 2011).

Recently, we have demonstrated that Hypericin (Hyp) has the potential to be used as a BH3 mimetic. Hyp is a naturally occurring compound in plants of the genus Hypericum and some endophytic fungi, such as the *Dermocybe* (Agostinis et al., 2002; Dewick, 2002; Miskovsky, 2002). It is a photosensitive phenanthroperylenquinone with the high quantum yield of singlet oxygen (Agostinis et al., 2002; Miskovsky, 2002). Hyp and its derivatives were the subjects of investigations for a long time due to their antitumor, antiviral and antidepressant properties (Miskovsky, 2002; Theodossiou et al., 2009; Berlanda et al., 2010; Karioti and Bilia, 2010; Kepp et al., 2014). The Hyp phototoxic effects have been extensively explored in normal and malignant mammalian cell lines, bacteria, and viruses (Theodossiou et al., 2009; Karioti and Bilia, 2010; de Andrade et al., 2021). There are extensive reviews published regarding Hyp subcellular localization and its potential cellular targets based on the cell death mechanisms triggered by the Hyp photodynamic action (Agostinis et al., 2002; Kiesslich et al., 2006; de Andrade et al., 2021). Currently, Hyp is used as a photosensitizer in photodynamic therapy (PDT) of tumors, inflammatory diseases, and infections (Miskovsky, 2002; Karioti and Bilia, 2010; de Andrade et al., 2021). Due to its hydrophobic properties, Hyp freely crosses the cell membrane and accumulates mainly in membranous organelles such as ER, mitochondria, Golgi apparatus and lysosomes (Rizzuto et al., 1998; Annis et al., 2001; Gajkowska et al., 2001; de Brito and Scorrano, 2010; Balogova et al., 2013). It has been claimed that Hyp and its derivates are compounds with a minimal cytotoxicity prior to illumination (Miskovsky, 2002; Theodossiou et al., 2009). However, there is growing evidence to contrary, including our recent work (Martinez-Poveda et al., 2005; Gyenge et al., 2013; Larisch et al., 2013; Larisch et al., 2014; Huntosova et al., 2017; Stroffekova et al., 2019). It has been shown that Hyp can accumulate in normal and tumor mammalian cells at a similar level and can display light-independent cytotoxicity in a wide range of concentrations (Blank et al., 2004; Mandel et al., 2007). Hyp also exhibited significant antiproliferative and anti-metastatic light-independent effects (Uzdensky et al., 2003; Noell et al., 2011). In the neuronal tissue, Hyp preferentially accumulated in glial and connective tissues including glioma tumors, whereas it entered into neurons at much lower level (Uzdensky et al., 2003; Noell et al., 2011).

In our recent work, we have shown, that Hyp presence in human endothelial and glioma cells resulted in significant light-independent effects on ultrastructure, mitochondria function and metabolism, and Bcl2 proteins’ distribution and synthesis (Huntosova et al., 2017). There are multiple molecular mechanisms that can explain Hyp light independent effects, for example, the reduction of intracellular pH, enzymes’ inhibition in cytoplasm, mitochondria or endoplasmic reticulum (ER) (Sureau et al., 1996; Gyenge et al., 2013; Dzurova et al., 2014), or interaction between Hyp and intracellular proteins (Agostinis et al., 1995; Agostinis et al., 1996; Halder et al., 2005; Lakowicz, 2006). However, these mechanisms are not well understood in detail and require more investigation. Based on Hyp accumulation in membranous organelles such as ER and mitochondria that are also primary activity sites for members of Bcl2 family (Rizzuto et al., 1998; Annis et al., 2001; Gajkowska et al., 2001; de Brito and Scorrano, 2010; Balogova et al., 2013) and our previous findings regarding Hyp effects on Bcl-2 protein distribution (Huntosova et al., 2017; Stroffekova et al., 2019), we explored the Bcl-2-Hyp interactions as one possible mechanism to explain Hyp light independent effects.

To summarize the above mentioned facts and findings, Hyp displayed effects at several sub-cellular levels. Hyp, like other known BH3 mimetics, played a key role in triggering apoptosis, caused changes in cellular ultrastructure, mitochondria function and metabolism, and in changed the distribution of Bcl-2 proteins in malignant and non-malignant cells (Huntosova et al., 2017). In addition, we have demonstrated that one of the mechanisms underlying these light independent effects can be a direct interaction between Hyp and Bcl-2 protein (Stroffekova et al., 2019).

In the present study, we tried to explore this interaction further. We have compare interaction of two BH3 mimetics, Gossypol (Goss) and ABT263, with the Bcl-2 protein BH1 and BH3 peptides and the whole purified Bcl-2 and Mcl-1 proteins to the Hyp interaction with these peptides and proteins. In addition, we explored the effects of these compounds on the viability of human glioma U87 MG cells and on the expression levels of Bcl-2 and Mcl-1 proteins. We have found that Hyp interacts more strongly with Bcl-2 and less with Mcl-1 protein than ABT263 or Goss. Incubation of U87 MG cells with Hyp and Goss downregulated Bcl-2 and upregulated Mcl-1 proteins. Hyp and ABT263 affected U87 MG viability insufficiently, where Goss decreased viability up to 20% in time and concentration manner. This suggests, that Hyp is interacting preferably with Bcl-2 protein similarly to ABT263, and Mcl-1 upregulation rescues viability of U87 MG cells. Further, we explored the use of Hyp and Goss combination treatment. In this treatment, we were able to reduce Goss concentration to reach a significant decrease in U87 MG viability. This finding indicates that Hyp can be explored in the drug combination therapy, however, more detailed study regarding this fact is required.

## Materials and methods

### Chemicals and reagents

An immortal cell line culture of malignant U87 MG human glioma cells was purchased from Cell Lines Services (CLS, Germany). Dulbecco´s modified Eagle medium (D-MEM) with high glucose (4,500 mgL^−1^) was obtained from ThermoFisher (Slovakia) and FBS was purchased from Sigma Aldrich (Slovakia). Hyp was purchased from Gibco-Invitrogen (France). 3-(4,5-Dimethyl-2-thiazolyl)-2,5-diphenyl-2H-tetrazolium bromide (MTT reagent), phosphate-buffered saline solution (PBS) and Dimethyl sulfoxide (DMSO) were obtained from Sigma Aldrich (Slovakia). ABT263, Gossypol, and purified whole Bcl2 and Mcl1 proteins were purchased from Abcam (Slovakia). Bcl2 BH1 and BH3 peptides were custom made by GeneCust (France) in HPLC purity (≥80%). Sodium chloride NaCl, Triton X-100, sodium deoxycholate, sodium dodecyl sulfate SDS, Tris, and Laemmli buffer were purchased from Sigma-Aldrich (Slovakia). Halt™ Protease and Phosphatase Inhibitor Cocktail was obtained from ThermoFisher Scientific (Slovakia).

Cell culture protocol - U87 MG cells were plated and maintained according to propagation protocols onto 35 mm Petri culture dishes (SPL, Switzerland). The cells grew in the dark as monolayer up to 80% confluence, at 37°C in a humidified 5% CO_2_ atmosphere. After reaching confluence, cells were incubated with either Hyp or Goss or their combination, and then processed in the MTT assay, or used for whole cell lysates in Western blot analysis.

### Hypericin and gossypol protocol

Hyp and Goss stock solutions were in dimethyl sulfoxide (DMSO) at concentration of 5 and 20 x10^−3^ M, respectively. Hyp and Goss were further diluted to final concentrations of 1, 5, 10, 15 and 30 µM in the cell culture medium. For all experiments, the final content of DMSO was less than 0.1%. Cells were incubated with either Hyp or Goss or their combination for 24 or 48 h in the dark in the presence of 5% CO_2_ humidified atmosphere at 37°C.

### MTT cell viability assay

U87 MG cells (2 × 10^3^ cells per well) were plated in 96-well plates. Appropriate culture medium was added to the each well, and cells were incubated at 37°C for the indicated times. MTT stock solution was at concentration 5 mg/ml in PBS. MTT was first diluted in fresh serum free medium (1:10) and then added into each well to a final volume of 100 μL. Yellowish MTT solution is converted to dark blue water-insoluble crystals of MTT formazan by mitochondrial dehydrogenases in live cells. Cells were then incubated for 2h in the dark (5% CO_2_, 37°C). Medium was then aspired and formazan crystals were dissolved in 100 μL DMSO per well. The absorbance of soluble formazan was measured with a microplate reader (GloMax, Promega) at 590 nm. Cell viability was displayed as the percentage of treated cells relative to the control ones. The EC50 values have been calculated by online tool Quest Graph™ EC50 Calculator (AAT Bioquest. https://www.aatbio.com/tools/ec50-calculator).

### Fluorescence spectra measurements

**FIGURE 1:**
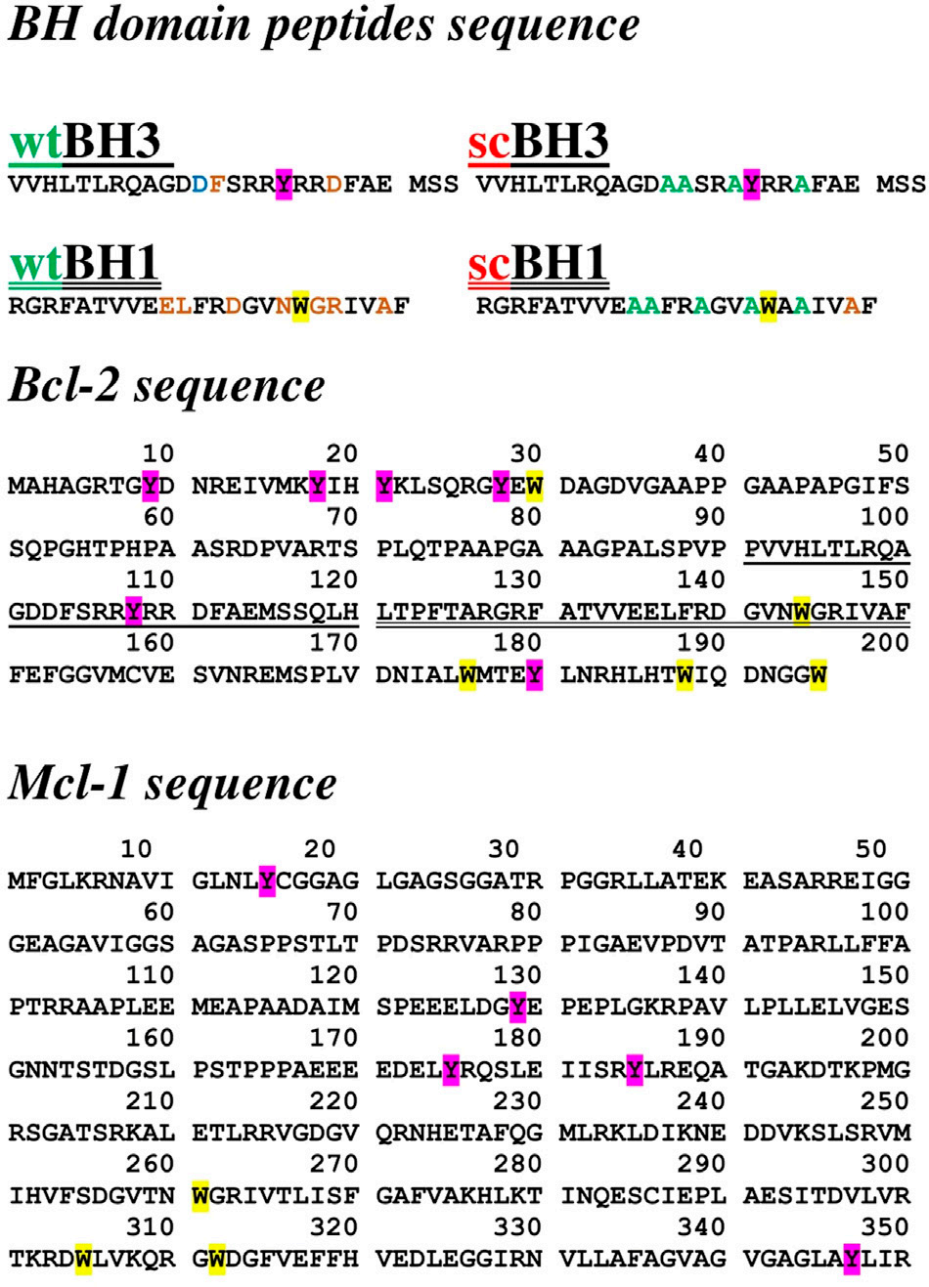
Amino acid sequences of Bcl2 BH1 and BH3 peptides, and Bcl2 and Mcl1 proteins.

Fluorescence spectra measurements were performed by a spectrofluorometer with 150W Xenon lamp (RF-5301 PC, Shimadzu, Japan). Samples were placed in a fluorimeter in the cuvette with volume 0.7 ml (Hellma 104.002-QS, Germany) and path length 10 mm. All spectra were recorded at room temperature, and in the range of wavelengths from 290 to 400 nm. Measurements were carried out in either DMSO or phosphate buffered saline solution (PBS) (pH 7.4) (Sigma Aldrich, Slovakia). Excitation wavelengths were 275 and 280 nm, respectively, which corresponded to the presence of tyrosine (Y) and tryptophan (W) residues in BH domain peptides and Bcl2 and Mcl1 proteins (Figure 1). Bcl2 protein BH1 (26 AA) and BH3 (24 AA) domain peptides were designed and described in detail previously (Stroffekova et al., 2019). In peptides with scrambled sequence, AAs important for ligand interaction were substituted with alanines (A). Peptide stocks were prepared at 2 mM concentration in DMSO. Hyp, Goss and ABT263 stock solutions were also prepared in DMSO at 100μM and 1 mM concentration. Final concentration of BH1 and BH3 peptides used in experiments was 1 μM in each sample.

The fluorimeter setting includes excitation wavelengths, interval, speed of measurements, excitation and emission bandwidths. The excitation wavelength for BH1-sc and BH1-wt was 280nm, for BH3-sc and BH3-wt was 275 nm. For the BH1 peptides measurements, excitation and emission bandwidths were 3 nm. In measurements with the BH3 peptides, excitation and emission bandwidths were 5 and 10 nm. In all measurements we have used 1 nm intervals and medium speed setting.

The final concentration of Bcl-2 and Mcl-1 protein used in measurements was 1 μM. Measurements were carried out in phosphate-buffered saline solution (PBS), pH7.4. The Bcl2 and Mcl1 proteins were tested in both excitation wavelengths at 280 and 275 nm that are inherent for tryptophan (W) and tyrosine (Y) fluorescence, respectively. The Bcl-2 protein displayed a stronger signal due to higher number of W in comparison with Mcl-1. Due to this fact, we have used 3 and 5 nm excitation and emission bandwidth for Bcl-2, and higher values of 5 and 5 nm for Mcl1. The Hyp, Goss, and ABT263 were added directly to a cuvette in increasing concentration ratio with proteins 1:1, 1:5, 1:10, 1:20, 1:30 μM. Fluorescence signals were analyzed by Origine software (OrigineLab, United States).

From the measured peptide/protein fluorescence spectra, we have derived the Stern–Volmer plots of F_O_/F versus [Q], where [Q] is the ligand concentration (Berlanda et al., 2010). The Stern-Volmer quenching constants K_d_ and the correlation coefficient of each curve were calculated from the slope of the regression curves using Eq (1).

$$\frac{F_{0}}{F}=1+K_{SV}\left[Q\right]=1+K_{d}\left[Q\right]=1+k_{q\;}\tau_{0}\left[Q\right]$$ (1)

In this equation, K_sv_ is the Stern-Volmer quenching constant, k_𝑞_ is the bimolecular quenching constant, τ_0_ is the lifetime of the fluorophore in the absence of quencher, and [Q] is the quencher concentration.

### Western blot (WB)

Confluent adherent U87 MG cells were washed 2X with ice-cold PBS. Subsequently, cells were lysed and homogenized in radioimmunoprecipitation (RIPA) buffer (150 mM sodium chloride, 1% Triton X-100, 0.5% sodium deoxycholate, 0.1% sodium dodecyl sulfate, 50 mM Tris, pH 8) with the inhibitor cocktail (1:100 dilutions, Halt™ Protease and Phosphatase Inhibitor Cocktail, respectively). The total protein content in the whole cell lysates was determined by the BCA protein assay (Pierce Chemical Co., Rockford, IL). For WB analysis, 20 μg of total protein per well was loaded onto 10% or 15% polyacrylamide gels and subjected to electrophoresis. The separated proteins were transferred to a nitrocellulose membrane (0.45 μm; Amersham Protran, Germany). Membranes were rinsed 3X with Tris-buffered saline with 0.1% Tween 20 (TBST) and then incubated with a blocking buffer. Mcl1, GAPDH and Bcl2 proteins in the membranes were blocked with 1% BSA and 1% dry nonfat milk in PBS, respectively, for 1 h at room temperature. After 3X wash with TBST, membranes were incubated overnight at 4°C with primary antibodies: anti-Bcl2 (1:100 dilution, ab196495, Abcam, Cambridge, UK), anti-Mcl1 (1:200, ab32087, Abcam, Cambridge, UK), and anti-GAPDH (1:2,500, ab9485, Abcam, Cambridge, UK) was used as loading control. Membranes were rinsed 3X with TBST and antibody bound proteins were detected and visualized using the Western Breeze Chromogenic Kit (ThermoFisher Scientific, Waltham, MA, United States). The protein band optical densities (O.D.) were analyzed by ImageJ software. After normalization to GADPH, the O.D. are presented in the histograms that represent the means of 3 measurements. The error bars represent the standard deviations.

### Statistical analysis

Experiments were repeated in at least three independent repetitions for all conditions. Statistical analysis was carried out by either Student’s t- test or ANOVA using SigmaPlot (Ver. 12.0; SystatSoftw. Inc.). A *p* < 0.05 was considered significant.

## Results

### Bcl2 BH peptides’ fluorescence spectra measurements

Previous research from our laboratory has shown that our designed and custom made peptides (Figure 1), corresponding to BH1 and BH3 domains of Bcl2 protein, interact with Hyp and known BH3 mimetic ABT263 (Stroffekova et al., 2019). Both, BH1 and BH3 peptides contain one endogenous fluorescent amino acid, tryptophan (W18) and tyrosine (Y17), respectively.

**FIGURE 2:**
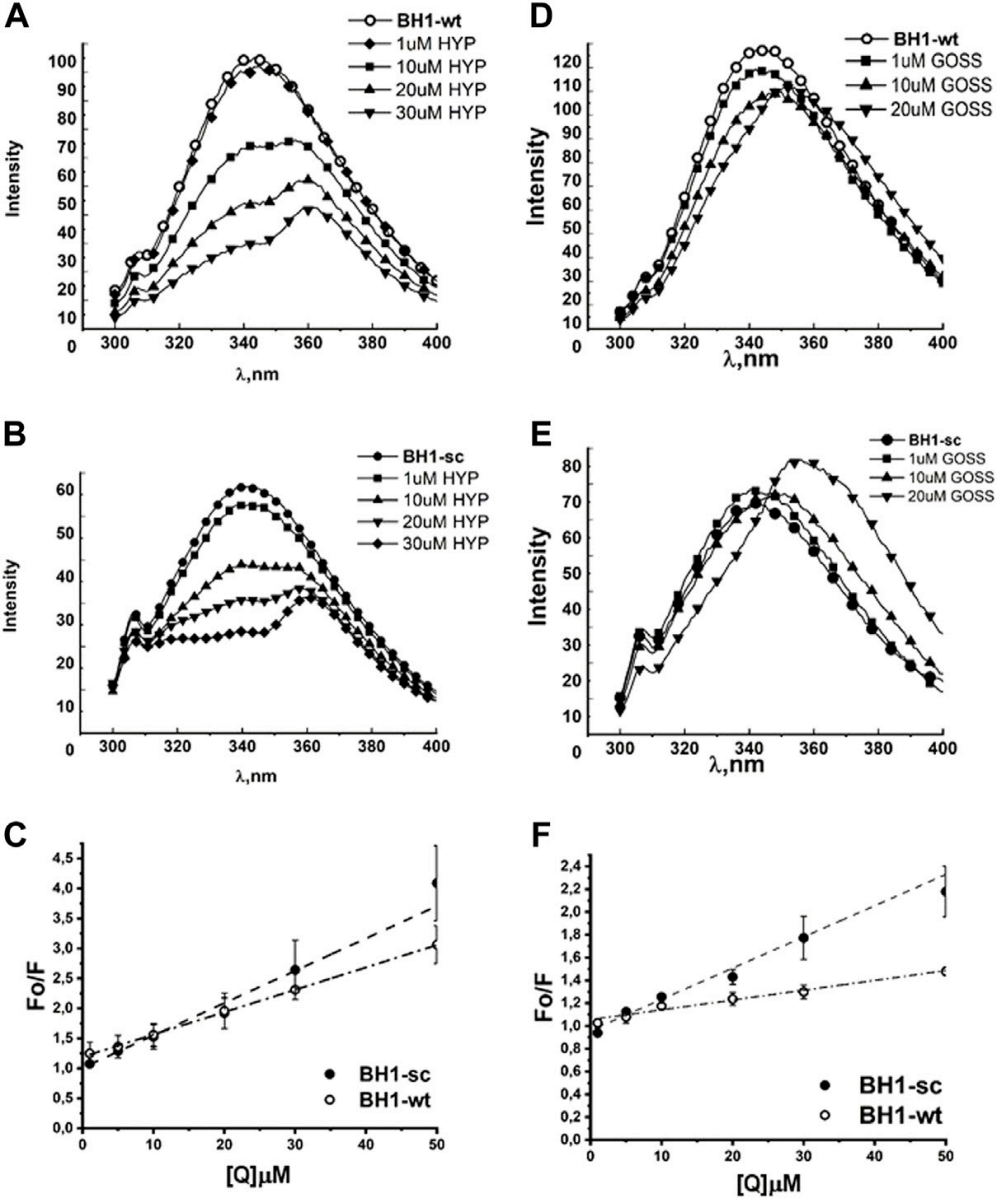
Hyp displays stronger quenching of BH1 peptides fluorescence spectra than Goss. The BH1-sc and BH1-wt fluorescence quenching spectra with different concentration of Hyp **(A,B)** and Goss **(D,E)**. **(C)** The linear fit of Stern-Volmer curves for BH1-sc and BH1-wt corresponding to quenching spectra in **(A,B)**. **(F)** The linear fit of Stern-Volmer curves for BH1-sc and BH1-wt corresponding to quenching spectra in **(D,E)**. All of the measurements were done in three independent measurements according to protocols described in Methods section.

**FIGURE 3:**
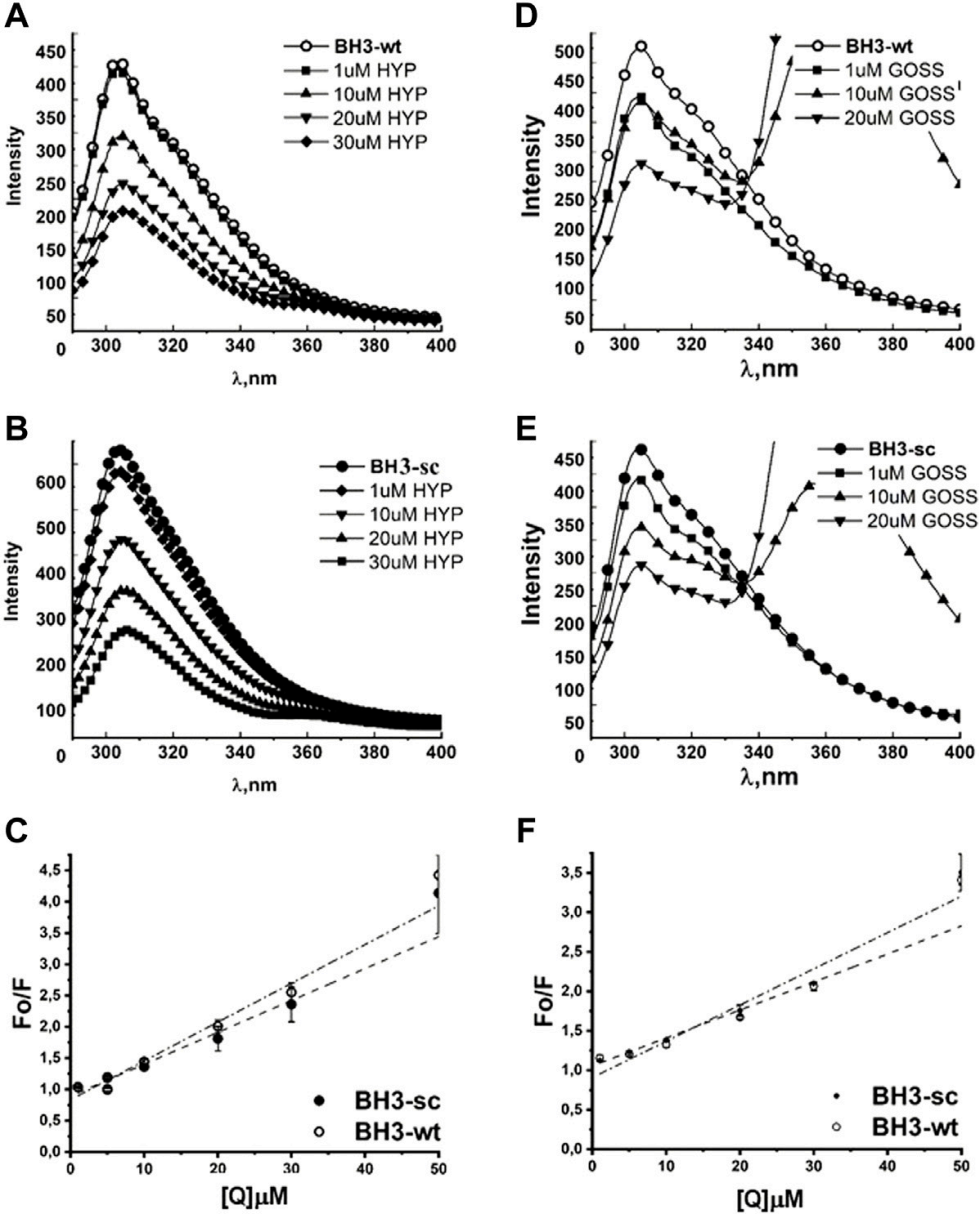
Hyp displays stronger quenching of BH3 peptides fluorescence spectra than Goss. The Fluorescence quenching spectra of BH3-sc and BH3-wt with different concentration of Hyp **(A,B)** and Goss **(D,E)**. **(C)** The linear fit of Stern-Volmer curves for BH3-sc and BH3-wt corresponding to quenching spectra in **(A,B)**. **(F)** The linear fit of Stern-Volmer curves for BH3-sc and BH3-wt corresponding to quenching spectra in **(D,E)**. All of the measurements were done in three independent measurements according to protocols described in Methods section.

Here, we show and compare the BH1 and BH3 peptides interactions with Hyp and another plant based BH3 mimetic, gossypol (Goss). We have studied changes in the fluorescence spectra of Figure 2) and BH3 peptides (Figure 3) with respect to the different concentrations of Hyp and Goss in DMSO. The BH1-wt and BH1-sc spectra in the absence of ligands display maximum intensity at 342 and 343 nm, respectively. These values correspond to a typical W maximum intensity in nonpolar environment.

Figure 2 shows the fluorescence emission spectra of BH1-wt and BH1-sc peptides in nonpolar solvent DMSO. Peptides were dissolved in DMSO at the final concentration of 1 μM. The BH1-wt and BH1-sc spectra were recorded within 300–400 nm range with 280 nm excitation wavelength. We observed increased W fluorescence quenching with increasing Hyp concentrations in both fluorescence spectra of BH1-wt and BH1-sc peptides (Figure 2A–C). At the high Hyp concentrations of 20 and 30μM, we observed red shift of the fluorescence maximum from 342 to 362 nm. In the presence of Goss, we observed W fluorescence quenching with increasing Goss concentrations in both fluorescence spectra of BH1-wt and BH1-sc peptides (Figure 2D, E). However, the level of quenching by Goss was clearly less pronounced than quenching by Hyp.

From the measured peptide fluorescence spectra, we have derived the Stern–Volmer curves of F_O_/F versus [Q] , as shown in peptides(Figure 2C, F), where [Q] is the Hyp and Goss concentration, respectively. The Stern-Volmer quenching constants $K_{d}$ (Table 1) and the correlation coefficient of each curve were calculated from the slope of the regression curves using Eq (1) in Methods.

**TABLE 1:** Stern-Volmer constants for BH1 and BH3 peptides interactions with Hyp or Goss.

$K_{d}$ x 10^12^ LM^-1^s^-1^		
Peptide	Hyp	Goss
BH1-wt	0.5447 ± 0.0000342	2.738 ± 0.00298
BH1-sc	0.405 ± 0.0000974	0.86 ± 0.000889
BH3-wt	0.493 ± 0.000409	3.546 ± 0.00258
BH3-sc	0.417 ± 0.000339	4.588 ± 0.00513

The correlation coefficients in the presence of Hyp and Goss were R = 0.99 and 0.98 for BH1-wt and BH1-sc, respectively. A typical fluorescence life time (τ_0_) of small biopolymers without the quencher is usually around 10^-8^s (Theodossiou et al., 2009; Karioti and Bilia, 2010). Using this value, we calculated Hyp quenching constants for our peptides at values 5.4 and 4.05 x 10^11^ LM^-1^s^-1^ for BH1-wt and BH1-sc, respectively. Further, we calculated Goss quenching constants for BH1 peptides at values 2.738 and 0.86 x 10^12^ LM^-1^s^-1^ for BH1-wt and BH1-sc, respectively.

Next, we tested BH3-sc and BH3-wt peptides interactions with either Hyp or Goss (Figure 3). In the absence of ligands, the BH3-wt and BH3-sc spectra display maximum intensity at 304 and 308 nm, respectively. These values correspond well with a typical tyrosine (Y) maximum intensity in nonpolar environment (Berlanda et al., 2010). In both BH3 peptides spectra, we observed a decrease in fluorescence intensity with increasing concentration of either Hyp (Figure 3A–C) or Gossypol (Figure 3D–F), which indicates interactions between peptides and used ligands. In the presence of Goss high concentration of 10–20 μM, we observed decreased tyrosine fluorescence and another fluorescence peak at maximum of 370nm, which is due to Goss itself (Supplementary Figure S1).

We have fitted the Stern-Volmer curves of measured spectra (Figure 3C, F). The Stern-Volmer quenching constants K_d_ derived from linear fits of BH3-wt and BH3-sc peptides with either Hyp or Goss are in Table 1. The correlation coefficients for Hyp interaction with peptides were R = 0.966 and 0.968 for BH3-wt and BH3-sc, respectively. The correlation coefficients for Goss interaction with peptides were R = 0.97 and 0.98 for BH3-wt and BH3-sc, respectively. Further we calculated quenching constants for Hyp and Goss. Hyp quenching constants were determined at values 4.93 and 4.17 x 10^11^ LM^-1^s^-1^ for BH3-wt and BH3-sc, respectively. Goss quenching constants were determined at values 3.54 and 4.59 x 10^12^ LM^-1^s^-1^ for BH3-wt and BH3-sc, respectively.

Next, we have compared our results from fluorescence emission spectra of BH1 and BH3 peptides with the predicted strength of interaction from our *in silico* experiments (Stroffekova et al., 2019). The predicted strength of interactions based on QPLD docking (Stroffekova et al., 2019) and results from fluorescence emission spectra of BH1 and BH3 peptides obtained here and in (Stroffekova et al., 2019) are in Table 2.

**TABLE 2:** Comparison of predicted and measured strength of interaction between BH peptides and selected BH3 mimetics.

Peptid	Predicted strength of interaction	Measured strength of interaction by fluorescence quenching
wBH1	Goss ˃ Hyp ˃ABT-263	Hyp ˃ABT-263 ˃ Goss
scBH1	Goss ∼ ABT-263 ˃ Hyp	ABT-263 ˃ Hyp ˃ Goss
wBH3	Goss ˃ Hyp ∼ ABT-263	ABT-263 ˃ Hyp ˃ Goss
scBH3	Goss ˃ ABT-263 ˃ Hyp	ABT-263 ˃ Hyp ˃ Goss

### Bcl2 and Mcl1 proteins’ fluorescence spectra measurements

In the next step, we have investigated interaction of known BH3 mimetics, Goss and ABT263, and Hyp in more physiologically relevant situation. We studied the interaction of these compounds with whole purified Bcl2 and Mcl1 proteins in the phosphate buffered saline (PBS) solution atpH 7.4. Figure 1 shows amino acid sequences of used BH peptides as well as Bcl2 and Mcl1 proteins, which we used in our experiments. Both, BH1 and BH3 peptides contain only one fluorescent amino acid, tryptophan (W) and tyrosine (Y), respectively. In contrast, whole proteins contain several fluorescent amino acid residues. Bcl2 protein contain six Y and five W residues, where Mcl1 protein has five Y and only three W residues. Therefore, we had measured fluorescence spectra of both proteins with excitation wavelength 275 and 280 nm. This gave us opportunity to see whether the changes in the spectra depends more on the emission from W or Y.

**FIGURE 4:**
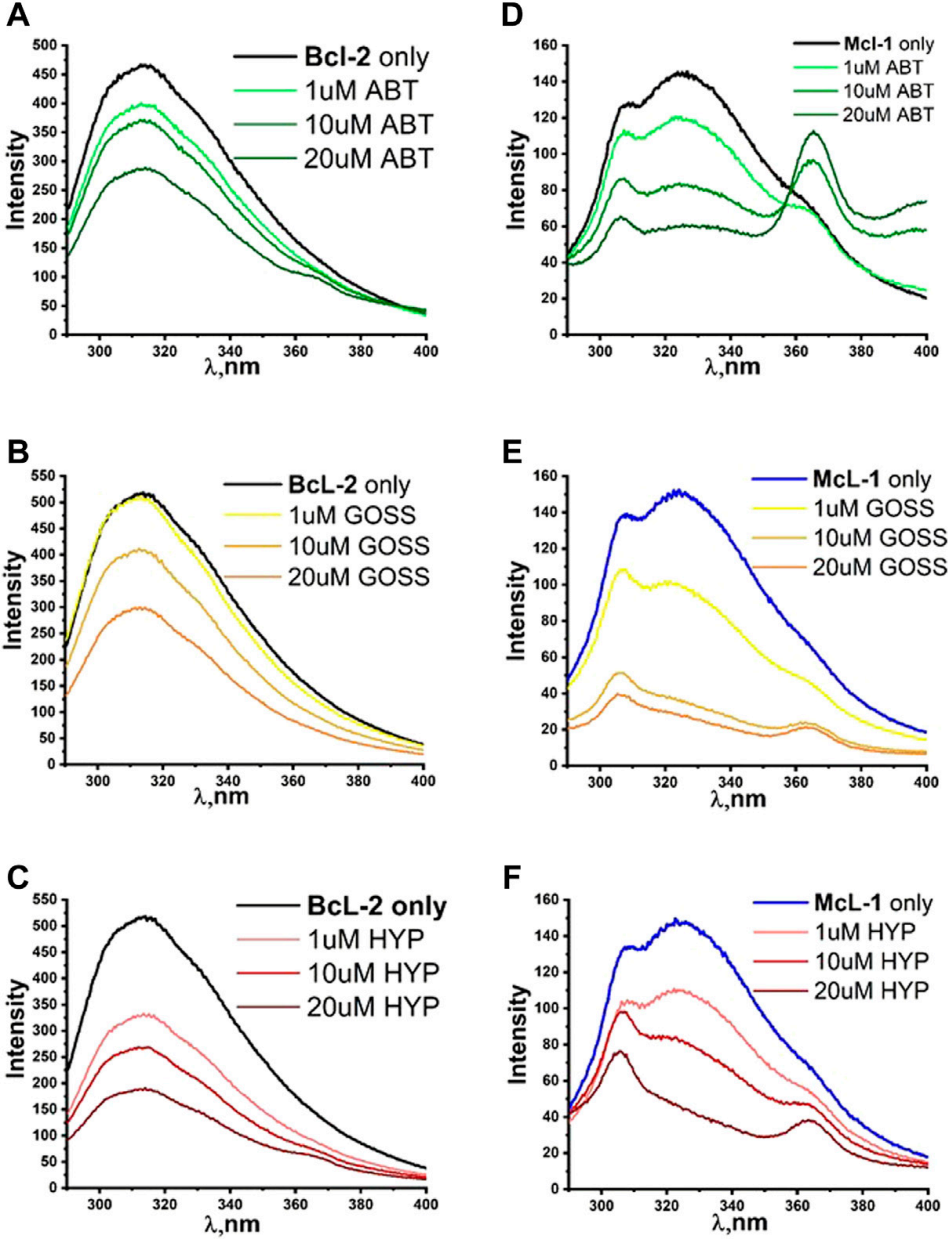
The fluorescence quenching spectra of Bcl2 and Mcl1. The fluorescence quenching spectra of Bcl2 **(A–C)** and Mcl1 **(D–F)** proteins (1 μM) with different concentration of ABT263, Goss and Hyp in PBS. Bcl2 spectra were measured with excitation wavelength of 280 nm and Mcl1 spectra with excitation wavelength of 275nm.

Figure 4 shows the fluorescence quenching spectra of Bcl2 and Mcl1 proteins at 1 μM concentration with different concentration of selected BH3 mimetics Hyp, Goss and ABT-263. Bcl2 protein spectra measured with excitation wavelength either 275 or 280 nm displayed spectra with maximum at 318±2 nm corresponding to combination of signals originated in Y and W residues, respectively. Here, we show data measured with 280 nm excitation (Figure 4A–C). In Bcl2 protein spectra, we have observed fluorescence signal quenching with increasing compound concentrations, with the strongest quenching byby Hyp.

**FIGURE 5:**
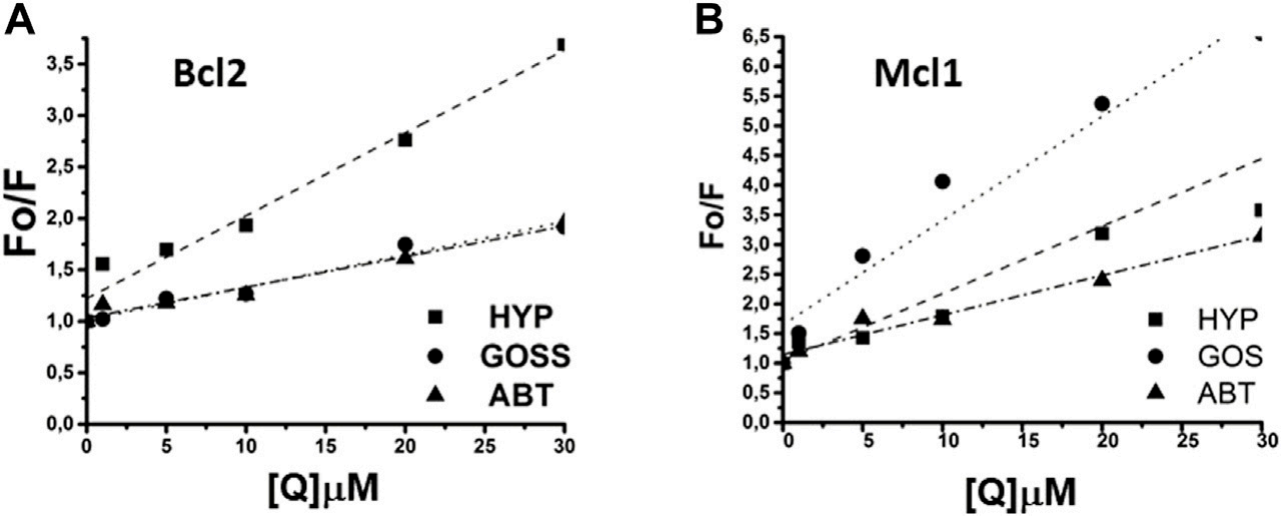
The linear fit of Stern-Volmer curves for Bcl2 and Mcl1 proteins. **(A)** The linear fit for Bcl2 and **(B)** for Mcl1 protein interaction with Hyp, Gossypol, and ABT263.

The Mcl1 protein spectra measured with excitation wavelength either 275 or 280 nm displayed spectra with two distinctive peaks at 308±2 and 323±2 nm corresponding to signals originated in Y and W residues, respectively. Here, we show data measured with 275 nm excitation (Figure 4D–F), where the distinction of two peaks was more pronounced. In Mcl1 protein spectra, we have observed fluorescence signal quenching with increasing compound concentrations, with the strongest quenching byGoss. Further, we have also fitted the Stern-Volmer curves of measured spectra (Figure 5). The Stern-Volmer quenching constants K_d_ derived from linear fits of Bcl2 and Mcl1 with ligands are in Table 3. The correlation coefficient for compound interaction with Bcl2 protein was 0.98 for all ligands. The correlation coefficient for compound interaction with Mcl-1 protein was 0.98 for Hyp and ABT-263, and 0.97 for Goss.

**TABLE 3:** The Stern-Volmer constants for Bcl2 and Mcl1 proteins with Hyp, Gossypol, and ABT263.

K_d_ x 10^14^ LM^-1^s^-1^			
Protein	Hyp	Goss	ABT263
Bcl2 (280nm)	0.08048±0.007	0.03191±0.00264	0.02971±0.00258
Mcl1 (275nm)	0.08825±0.00888	0.17994±0.00206	0.06566±0.00622

Next, we have compared the predicted strength of interaction between Bcl2 or Mcl1 and selected BH3 mimetics based on the QPLD docking (Stroffekova et al., 2019) with the results from the fluorescence quenching measurements. The measured strength of interaction differs from the predicted order.

### The Hyp and Goss cytotoxicity in U87 MG cells

To analyze whether inhibition of anti-apoptotic Bcl-2 proteins is sufficient to induce glioma cell death, we used two different BH3 mimetics, Hyp and Goss, in this study. Based on our fluorescent quenching measurements with the BH peptides and whole Bcl2 and Mcl1 proteins, we concluded that Hyp and Goss interact with these proteins.

**FIGURE 6:**
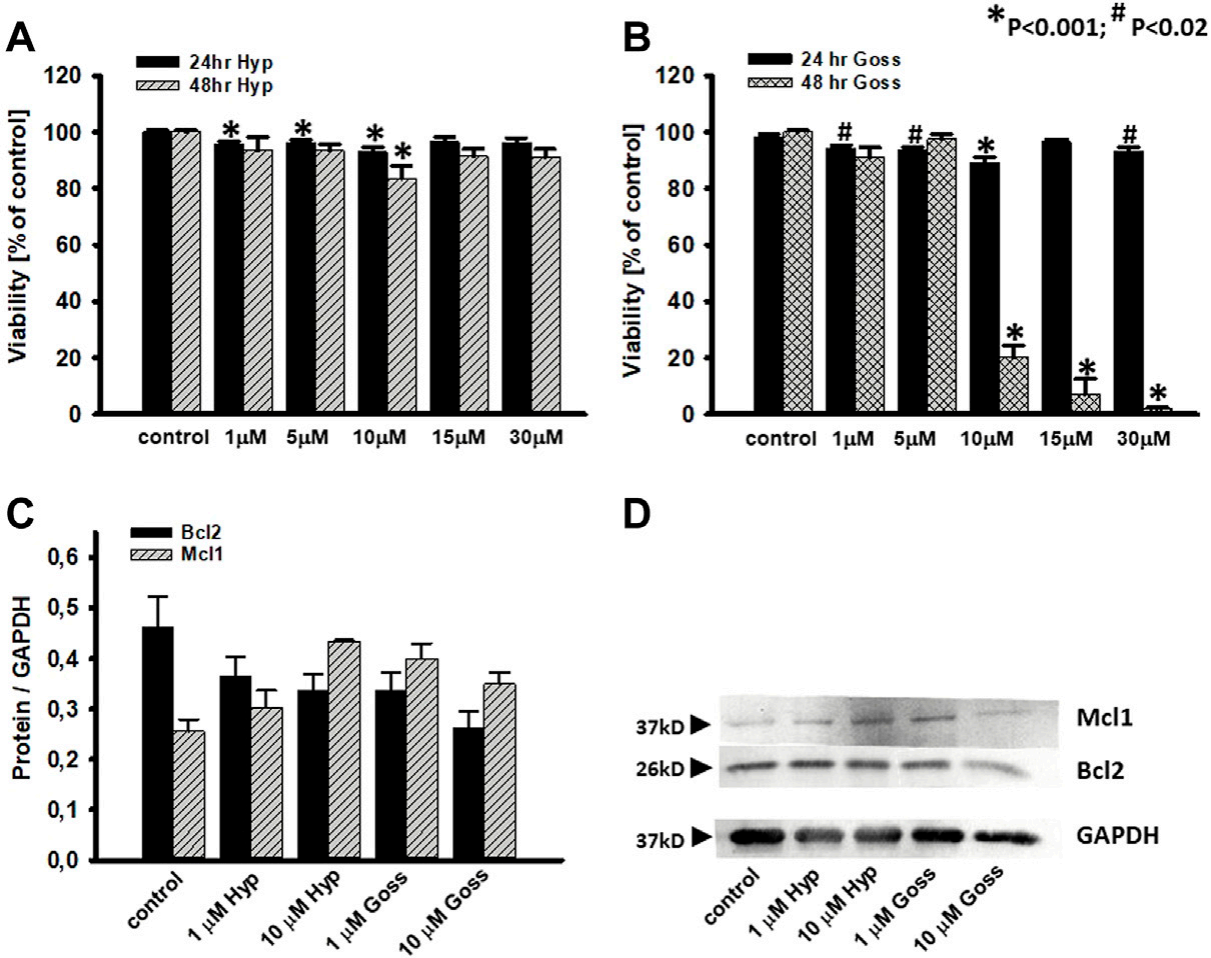
Effects of Hyp or Goss on U87 MG cells viability and Bcl2 and Mcl1 protein levels. **(A)** Cell viability in the presence of Hyp and **(B)** Goss for 24 and 48 h. **(C)** The optical density band histograms of Bcl2 and Mcl1 normalized against GAPDH after 48 h incubation with Hyp or Goss. The histograms represent average of three independent experiments and the error bars represent the standard deviations from the average values. All experiments were repeated in triplicates. **(D)** Expression of Mcl1 and Bcl2 proteins in U87 MG whole cell lysates after 48 h incubation with Hyp or Goss was examined by western blot analysis. GAPDH was used as a housing protein.

To test these interactions further, we have tested cell viability in the presence of Hyp and Goss in human U87 MG glioma cell line by MTT assay. We have used Hyp and Goss in concentration 1, 5, 10, 15, 30 μM. U87 MG cell viability after 24 and 48h incubation of cells with either Hyp or Goss is shown in Figure 6. In all used concentrations, Hyp after 24h slightly decreased cell viability by 5–7% at 1–10 μM Hyp (Figure 6A). However, this decrease is not sufficient to render the compound cytotoxic. After 48h treatment, there was no significant effect on cell viability except for 10 μM concentration of Hyp, where we detected decrease in viable cells to 78% (Figure 6A, *p* < 0.021). Incubation of U87 MG cells with Goss for 24h resulted in the similar effect on viability as with Hyp (Figure 6B). Goss after 24h slightly decreased cell viability by 6–10% at 1–10 μM Goss. In contrast, after 48h Goss caused significant decrease (*p* < 0.0001) in cell viability below 20% at concentration above 10 μM (Figure 6B). The EC50 for Goss (48h) has been determined at 8.45 μM. Consequently, Goss exhibited stronger effect on U87 MG cell viability that Hyp.

To explore this further, we have also investigated Mcl1 and Bcl2 protein level of expression in U87 MG cells after 24 and 48h incubation with either Hyp or Goss (Figure 6C, D). Hyp and Goss caused downregulation of Bcl2 and upregulation of Mcl1 protein in time and concentration dependent manner, where 10 μM Hyp (48h) caused the 1.7fold increase of Mcl1.

**FIGURE 7:**
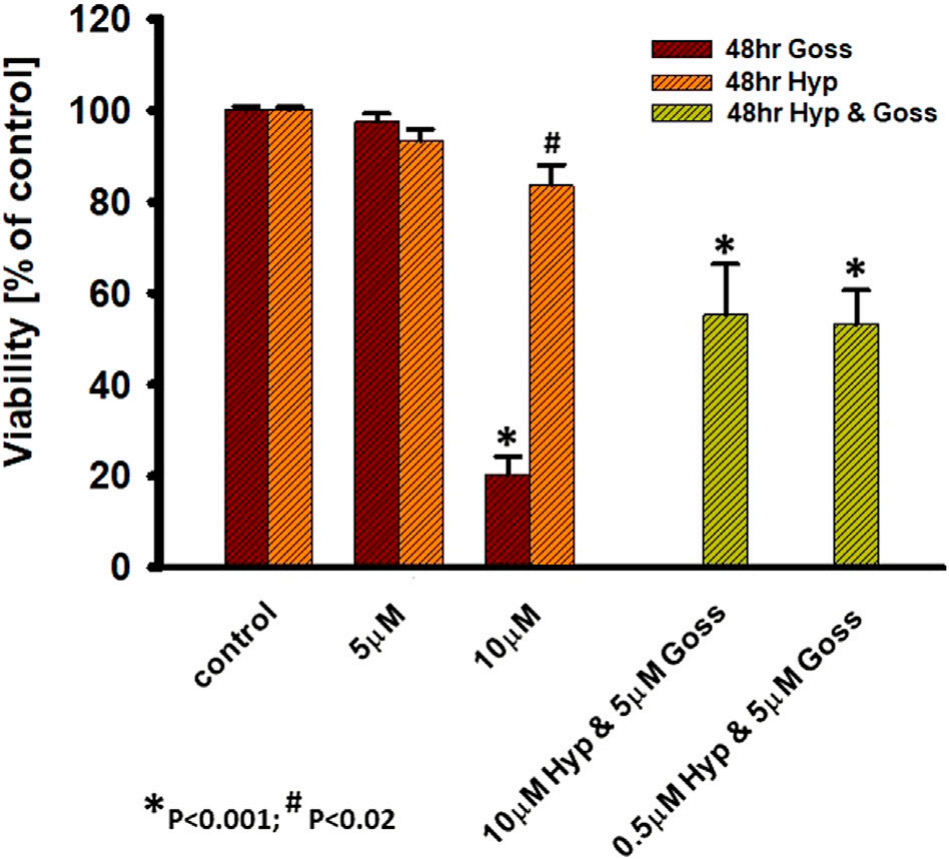
Hyp and Goss have synergetic effect on U87 MG cells viability. Cell viability in the presence of Hyp and Goss for 48 h.

In the next step, we did pilot experiments regarding the possibility of Goss and Hyp combined effect. We used the subthreshold Goss concentration of 5 μM and used two Hyp concentrations 0.5 and 10 μM (Figure 7). In comparison with the 10 μM Hyp alone, presence of 5 μM Goss significantly decreased cell viability to 55%. In addition, even low 0.5 μM concentration of Hyp in combination 5 μM Goss resulted in the significant decrease in cell viability to 53%, where 5 μM Goss alone did not. These results indicate a possible synergy between two compounds.

## Discussion

In various examples of human cancer lines such as prostate cancer, myeloid leukemia, breast cancer, glioblastoma etc., it has been demonstrated that they have heighten expression of multiple anti-apoptotic Bcl-2 proteins (Bcl-2, Bcl-XL and Mcl-1) and have displayed strong chemo resistance (Wei et al., 2011; Sadahira et al., 2014; Vogler, 2014; Correia et al., 2015; Gong et al., 2016; Frame et al., 2020). It has been also demonstrated that inhibition of only one anti-apoptotic member in these cells is not sufficient to trigger apoptosis. The various Bcl-2/Bcl-XL inhibitors, including ABT263, upregulated Mcl1 in malignant cells and rendered them resistant to these compounds (Wei et al., 2011; Zhao et al., 2013; Wang et al., 2014; Lee et al., 2018). The upregulated Mcl1 can also inhibit autophagy, which could augment apoptosis in cancer cells (Maiuri et al., 2007; Voss et al., 2010). Therefore, it is of interest to explore new possible BH3 mimetics and to test them in the combination with known Bcl-2 proteins inhibitors or other cancer treatments to achieve possible synergy.

In this work, we have investigated further the interaction of Hyp, a naturally occurring compound, with anti-apoptotic Bcl-2 family members Bcl-2 and Mcl-1. As shown earlier, Hyp interacted with BH1 and BH3 peptides of Bcl-2 protein similar to known Bcl-2 inhibitor ABT263, and our QPLD docking data indicated that Hyp could interact with Bcl-2 and Mcl-1 similar to pan-Bcl-2 BH3 mimetic, Goss (Stroffekova et al., 2019).

First, we investigated interactions of BH domain peptides from Bcl-2 protein, BH1 and BH3, with Hyp and Goss. BH1 and BH3 domain are part of a hydrophobic pocket in Bcl-2 proteins, which play an important role in the interaction between anti- and pro-apoptotic Bcl-2 proteins. These domains are also part of the interacting site for BH3 mimetics (Wang et al., 2000b; Wang et al., 2006). We have studied changes in the fluorescence spectra of BH1 (Figure 2) and BH3 peptides (Figure 3) with respect to the different concentrations of Hyp and Goss in DMSO. We observed increased W fluorescence quenching with increasing Hyp and Goss concentrations in both fluorescence spectra of BH1-wt and BH1-sc peptides (Figure 2). These findings are in good agreement with our previous results (Stroffekova et al., 2019), and further confirm Hyp interaction with the BH1 peptides. We have observed that the level of quenching by Goss was clearly less pronounced than quenching by Hyp, indicating that Hyp interaction with BH1 peptides is stronger. At the high Hyp concentrations of 20 and 30μM, we have noticed a red shift of the maximum fluorescence peak to 362 nm. The red shift in spectra may be observed due to several reasons such as change in hydrogen bonds, dipole moment or change of solvent (Kepp et al., 2014). In our case, where we increased Hyp concentration, the reason for the red shift may be caused by increased hydrogen bonds between Hyp and peptide.

The BH3-wt and BH3-sc peptide fluorescent spectra (Figure 3) display maximum intensity at 304 and 308 nm, respectively, which correspond well with a typical Y maximum intensity in nonpolar environment. Similar to observation with BH1 peptides, we have noticed a fluorescence quenching with increasing concentration of either Hyp or Goss, which indicates interactions between peptides and used ligands. In the presence of Goss high concentration of 10–20 μM, we observed decreased tyrosine fluorescence and another fluorescence peak at maximum of 370nm, which is due to Goss itself (Supplementary Figure S1). The Stern-Volmer quenching constants $K_{d}$ (Table. 1) derived from the BH1 and BH3 peptides fluorescent spectra for Hyp and Goss are at values larger than the limiting diffusion rate constant of the biomolecule (∼10^10^ LM^-1^s^-1^), and therefore they indicate the static quenching and some type of binding interaction (Shea et al., 1997; Lakowicz, 2006; Muino and Callis, 2009).

The findings from the BH1 and BH3 peptides helped us to determine the best concentration range of Hyp, ABT263 and Goss for investigating their interaction with Bcl-2 and Mcl-1 proteins. Figure 4 shows the fluorescence quenching spectra of Bcl-2 and Mcl-1 protein (1 μM) with different concentration of selected BH3 mimetics, Hyp, Goss and ABT-263. In Bcl-2 spectra, we have observed fluorescence signal quenching with increasing compound concentrations of all compounds, with the strongest quenching by Hyp. The decrease in fluorescence intensity with increasing concentration of compounds strongly indicates interactions between Bcl-2 protein and used ligands. In the case of Bcl-2 protein, Hyp seemed to have the strongest effect, followed by Goss and ABT-263, which seem to have similar strength of interactions. In Mcl-1 spectra, we have observed fluorescence signal quenching with increasing compound concentrations, with the strongest quenching by Goss. In spectra measured with ABT-263, we have observed additional peak of 364±2 nm at high ABT263 concentrations, which corresponds to ABT-263 fluorescence (Supplementary Figure S1). The decrease in fluorescence intensity with increasing concentration of compounds strongly indicates interactions between Mcl-1 protein and used ligands. In case of Mcl-1 protein, Goss seemed to have the strongest effect, followed by Hyp and then ABT263.

We have compared obtained results from the fluorescence quenching measurements with the predicted strength of interaction between Bcl-2 or Mcl-1 and selected BH3 mimetics based on the QPLD docking (Stroffekova et al., 2019). It is clear that predicted values differ from the measurements. The differences may be rooted in the polarity of the environment and differences in the protein structure used in the QPLD docking and actual protein structure in PBS.

In the next step, we tested the response of human U87 MG glioma cells to the treatment by Hyp and Goss alone, and their combination. Hyp did not substantially affected cell viability either at 24 or 48h incubation (Figure 6). This finding is in good agreement with our previously published results (Huntosova et al., 2017), and it suggests that Hyp did not inhibit all of the anti-apoptotic Bcl-2 proteins in U87 MG cells. This is similar to our findings that U87 MG cells show a significant resistance to ABT263 (Stroffekova et al., 2019). In contrast, Goss, known panBcl-2 inhibitor (Kiesslich et al., 2006), caused significant effect (*p* < 0.0001) after 48h on cell viability at concentration of 10 μM and higher. The cell viability decreased below 10% (Figure 6). These findings are in good agreement with published information regarding the Goss cytotoxicity (Shea et al., 1997; Sadahira et al., 2014). The difference between U87 MG sensitivity towards Hyp, ABT263 and Goss may reside in upregulation of anti-apoptotic Mcl-1 (Zhao et al., 2013; Wang et al., 2014; Lee et al., 2018) and/or Bcl-XL proteins (Zhao et al., 2013; Wang et al., 2014; Kotschy. et al., 2016; Caenepeel et al., 2018; Lee et al., 2018; Seiller et al., 2020). To elucidate the effects of Hyp and Goss further, we have looked at the Mcl-1 and Bcl-2 protein level of expression in U87 MG cell (Figure 6), and have found that Hyp and Goss downregulated Bcl-2 and upregulated Mcl-1 proteins. The upregulated Mcl-1 and different affinities of Hyp and Goss towards Mcl-1 may underlie different U87 MG sensitivity towards Hyp and Goss. In addition, differences may also be caused by off-target effects such as ER stress, increased oxidative stress and induction of autophagy, which were shown for many BH3 mimetics, including ABT737, gossypol, and obatoclax (Voss et al., 2010; Opydo-Chanek et al., 2017). The cytotoxicity of BH3 mimetics and their effects depend on the framework of expressed anti- and pro-apoptotic Bcl-2 proteins, and on off-target mechanisms involving activation of alternative cell death modes and modulation of multiple signaling pathways. There are still numerous questions regarding the pharmacological effects of BH3 mimetics that contribute to their cytotoxic activity (Vela and Marzo, 2015).

One of the possible strategies to overcome the cancer cell resistance towards Bcl-2 or Mcl-1 inhibitors is to use combination of both inhibitors (Seiller et al., 2020). We have tested combination treatment of Hyp and Goss on U87 MG cells. We have found that low 0.5 μM concentration of Hyp in combination 5 μM Goss resulted in a significant decrease in cell viability to 53% (Figure 7), suggesting that combination treatment is effective and enables to use low doses of compounds. Further, this pilot results suggest indicate a possible synergy between two compounds, however there have to be more detailed study of this phenomena and underlying mechanisms.

## Conclusion

In this work, we have investigated interactions of BH domain peptides from Bcl-2 protein, BH1 and BH3, with Hyp and Goss. By fluorescence quenching method, we have shown that Hyp and Goss interact with BH1 and BH3 peptides in concentration dependent manner. Hyp displayed stronger fluorescent quenching in both peptides than Goss indicating stronger Hyp interaction. Further, we have compared the interactions of known BH3 mimetics, Goss and ABT-263, with whole purified proteins, Bcl-2 and Mcl-1 to those of Hyp. Hyp also displayed the stronger fluorescent quenching in the interaction with the Bcl-2 protein than ABT263 and Goss. In the interactions with the Mcl-1 protein, Goss displayed the strongest effect, followed by Hyp and ABT-263. These findings suggested that Hyp interacts with Bcl-2 proteins in similar manner as the other BH3 mimetics. In human glioma U87 MG cell line, Hyp cytotoxicity was low, similar to that of ABT263, where Goss exerted sufficient cytotoxicity. This suggests that Hyp acts primarily on Bcl-2, but not on Mcl-1 protein. In addition, Hyp and Goss downregulated Bcl-2 and upregulated Mcl-1 proteins in U87 MG cells. In combination therapy, low doses of Hyp with Goss effectively decreased U87 MG viability, suggesting a possible synergy effect. Overall, we can conclude that Hyp as BH3 mimetic acts primarily on Bcl-2 protein. Thus, it can be used to treat malignancies depending on Bcl-2 over-expression, or in combination with other BH3 mimetics, that target Mcl-1 or Bcl-XL proteins, in dual targeting therapy.

## Data Availability

The original contributions presented in the study are included in the article/Supplementary Materials, further inquiries can be directed to the corresponding author.
