# Supplementary material for: Hypericin, a potential new BH3 mimetic
Source: Front Pharmacol. 2022 Oct 4;13:991554. doi: 10.3389/fphar.2022.991554 (PMC9577225; doi:10.3389/fphar.2022.991554)
Supplement: Supplementary file 1 [file DataSheet1.docx]

Supplementary Material
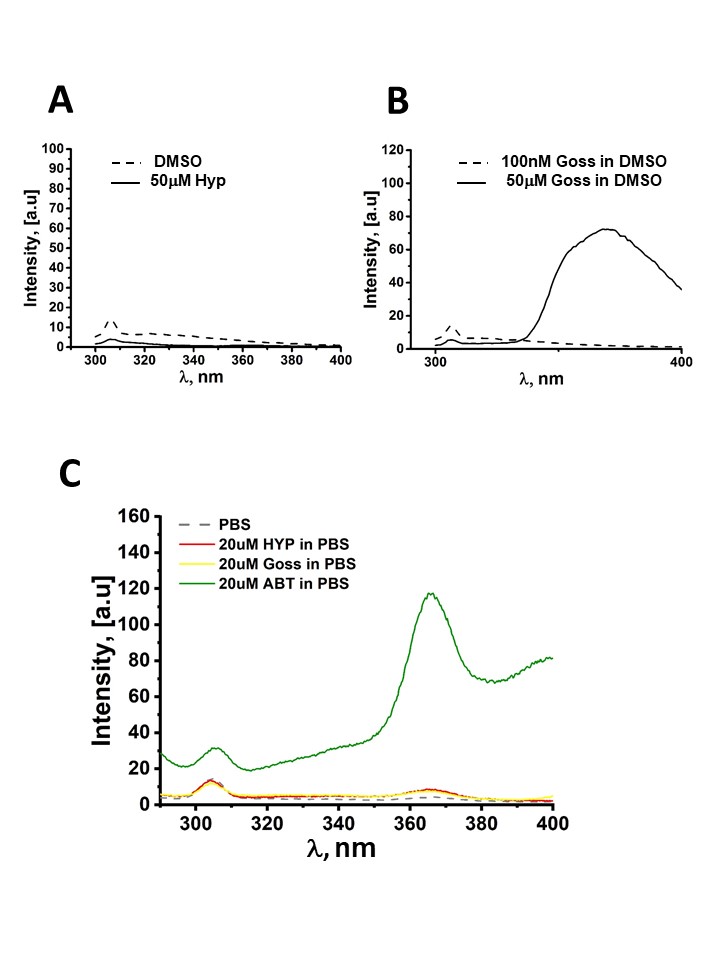


Supplementary Figure 1. Fluorescence spectra of the ligands alone. (A) Fluorescence spectra of Hyp alone in DMSO. (B) Fluorescence spectra of Goss alone in DMSO. (C) Fluorescence spectra of Hyp, Goss and ABT-263 alone in PBS.
